# Evaluation of recognition and signalling receptors on the peripheral blood cells of septic patients and their correlation with clinical outcomes

**DOI:** 10.1186/cc12664

**Published:** 2013-06-19

**Authors:** SC Silva, GL Baggio-Zappia, MKC Brunialti, E Silva, LCP Azevedo, FR Machado, R Salomao

**Affiliations:** 1 Hospital Sao Paulo, Escola Paulista de Medicina, Universidade Federal de Sao Paulo, SP, Brazil; 2 Hospital Israelita Albert Einstein, Sao Paulo, SP, Brazil; 3 Hospital Sirio Libanes, Sao Paulo, SP, Brazil

## Introduction

Monocytes and neutrophils play a key role in host defence by sensing and destroying microorganisms [1]. We evaluated the expression of cellular receptors implicated in pathogen recognition, cell activation and migration on both cell types during sepsis. Blood samples were collected from 77 septic patients (SP) at admission (D0), from 45 patients after 7 days of therapy (D7) and from 40 healthy volunteers (HV).

## Results

**Figure 1:**
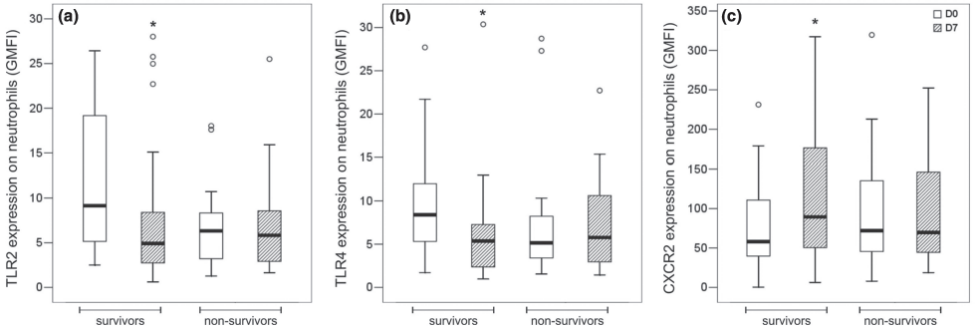
**Analysis of the expression of TLR2 **(A)**, TLR4 **(B) **and CXCR2 **(C) **surface receptors on neutrophils from the whole blood of surviving septic patients and nonsurvivors at D0 and D7**. Histograms and expressed as the geometric mean fluorescence intensity (GMFI). *P <0.05.

The expression of CD14 on monocytes and of CD11b and CXCR2 on neutrophils from SP was lower than that from HV. Conversely, the expression of TLR5 on monocytes and neutrophils was higher in SP when compared with HV. The expression of TLR2 on the surface of neutrophils and that of TLR5 on monocytes and neutrophils of SP was lower at D7 than at D0. In addition, the SP that survived showed reduced expression of TLR2 and TLR4 on the surface of neutrophils at D7 compared with D0 (Figure 1). The expression of CXCR2 for surviving patients was higher at follow-up when compared with baseline (Figure 1).

## Conclusion

The expression of recognition and cell signalling receptors is differentially regulated between SP and HV depending on the receptor being evaluated. However, despite these changes, it is likely that the functional changes in monocytes and neutrophils that are observed during sepsis are not directly linked to the modulation of the expression of TLRs [2].
